# Smart Beamforming for Direct LEO Satellite Access of Future IoT

**DOI:** 10.3390/s21144877

**Published:** 2021-07-17

**Authors:** Marius Caus, Ana Perez-Neira, Eduard Mendez

**Affiliations:** 1 Centre Tecnològic de Telecomunicacions de Catalunya (CTTC)/CERCA, 08860 Castelldefels, Spain; aperez@cttc.es; 2 Department of Signal Theory and Communications, Universitat Politècnica de Catalunya (UPC), 08034 Barcelona, Spain; eduardmendezlopez@gmail.com

**Keywords:** LEO, massive MIMO, massive IoT, beamforming, grant-free, orthogonal frequency division multiplexing, non-orthogonal multiple access

## Abstract

Non-terrestrial networks (NTN) are expected to play a key role in extending and complementing terrestrial 5G networks in order to provide services to air, sea, and un-served or under-served areas. This paper focuses the attention on the uplink, where terminals are able to establish a direct link with the NTN at Ka-band. To reduce the collision probability when a large population of terminals is transmitting simultaneously, we propose a grant-free access scheme called resource sharing beamforming access (RSBA). We study RBSA for low Earth orbit (LEO) satellite communications with massive multiple-input multiple-output (MIMO). The idea is to benefit from the spatial diversity to decode multiple overlapped signals. We have devised a blind and open-loop beamforming technique, where neither the receiver must carry out brute-force search in azimuth and elevation, nor are the terminals required to report channel state information. Upon deriving the theoretical throughput, we show that RBSA is appropriate for grant-free access to LEO satellite, it reduces the probability of collision, and thus it increases the number of terminals that can access the media. Practical implementation aspects have been tackled, such as the estimation of the required statistics, and the determination of the number of users.

## 1. Introduction

The communications market has experienced a steady growth in the last years, owing to the large number of devices connected to the Internet. The network that supports the connection of these devices leads to a system of interrelated machines, objects, or people, which gives rise to the well-known concept of Internet of things (IoT). In the coming era, it is estimated that there will be billions of devices connected globally. Industry organizations, such as GSMA, expect IoT connections to reach 25 billions by 2025, which is slightly more than double the 12 billion connections of 2019 [1]. In this context, non-terrestrial networks (NTN) emerge as a key technology to extend and complement terrestrial networks, which is crucial to deliver services any time and anywhere. Services can be provisioned in areas that are not covered by conventional infrastructures, such as fiber, cables, or radio frequency links. For instance, remote rural regions, deserts, and maritime areas, to mention a few. According to 3GPP, NTN include satellites located at different orbits as well as high altitude platforms [2].

In regards to the architecture, one of the possible applications of satellite communications is in the backhaul segment. In this deployment, terminals are indirectly connected to the satellite through a terrestrial node that aggregates the traffic. A more challenging architecture scenario considers the satellite in the radio access network (RAN), so that terrestrial infrastructures in the user link are not needed. This architecture allows direct access between the user equipment (UE) and the satellite. Establishing a direct link between the devices and satellite poses several challenges. In fact, typical satellite channel impairments, such as large path losses, delays, and Doppler shifts have to be assessed on radio protocols [3,4,5]. The orbit, frequency, and on-board digital processing usually dictate the severity of the adverse effects. Considering the large propagation loss and the long delay involved in geostationary Earth orbit (GEO) satellite communications, it follows that the most suitable option to allow direct access is to rely on non-GEO (NGEO), especially low Earth orbit (LEO), satellite communication systems. It is important to clarify that in regards to the delay, LEO is advantageous over GEO only if the LEO gateway coverage is global or if routing is allowed between LEO satellites by means of inter-satellite links (ISLs). Otherwise, LEO satellites need to adopt store and forward protocols, which significantly increase the delay. In such a situation, the network does not support real-time IoT services. It is worth emphasizing that there are two possible options of satellite payload implementations [2]. On the one hand, a transparent or bent-pipe satellite acts as a radio frequency repeater. In such a case, the satellite can be regarded as a flying relay node. On the other hand, the option considered in this paper is the regenerative transponder. With regenerative architecture, the satellite is able to generate/detect the signal to/from the devices.

The broadcast capabilities and the large footprint that characterize LEO satellite constellations can be harnessed to fit the needs of massive machine-type communications (mMTC), also referred to as massive IoT. In the satellite mMTC context, there are several challenges that deserve some attention. The main issue stems from the large population of devices that are located within the coverage of a single satellite. Indeed, the geographical area covered by a satellite is significantly higher than the size of typical terrestrial cells [6]. Hence, at a given instant, the number of concurrent users requesting access to the network through the satellite could yield congestion, unless some measures are taken. The problem of providing massive connectivity is addressed by employing multibeam technology and the non-orthogonal multiple access (NOMA) principle, where multiple users can overlap in the time-frequency plane. In the downlink, multiple non-orthogonal transmission schemes can be adopted to outperform orthogonal schemes [7]. Similarly, in the uplink it is possible to simultaneously serve several users on shared time and frequency resources. Note that scheduled transmission schemes, which involve close-loop signaling and multiple exchange of messages, are not suitable for low-duty IoT traffic. For the sporadic uplink transmission of multiple devices, the random access scheme is more efficient. To separate the messages in uplink NOMA, one can rely on the ideas highlighted in [8,9], where a comprehensive review of cellular and satellite access schemes are provided. Essentially, the concept is based on exploiting a codebook of signatures to facilitate user separation and detection. To exhibit a distinguishing feature, different operations, such as repetition, spreading, multi-dimensional modulation, interleaving, and scrambling can be employed. The sparse code multiple access (SCMA) [10] and the contention resolution diversity slotted Aloha (CRDSA) [11] are advanced access schemes that require time slot synchronization. Prominent access schemes that are able to operate in asynchronous mode include the asynchronous contention resolution diversity Aloha (ACRDA) [12], the enhanced spread spectrum Aloha (E-SSA) [13,14], and the asynchronous flipped SCMA (AF-SCMA) [15]. An alternative approach that is referred to as resource sharing beamforming access (RSBA) has been proposed in [16]. The innovative idea of the RBSA scheme, which is based on [17], stems from combining spatial signal processing and signature-based NOMA (S-NOMA) to achieve angular resolution. Then, it follows that terminals located in different spots can be separated and thus, the probability of collision is reduced. This scheme is blind as it does not require transmitting an specific training sequence to estimate the channel nor scanning in both elevation and azimuth. In this respect, the proposed RSBA is different from the ones that exist in the literature for terrestrial mMTC access with spatial diversity. In [18,19], the authors simplify the user detection problem by resorting to the channel hardening and favorable propagation properties of terrestrial massive multiple-input multiple-output (MIMO). However, this channel behavior is not present in satellite communications due to the strong line-of-sight (LoS) component. Another approach is that in [20], where, first, the channel must be estimated, and next, for each detected pilot sequence, a maximum ratio combination is applied to the data symbols. This procedure relies on averaging the pilot collision events across the transmission slots and is suitable for delay-tolerant and low-rate applications. In contrast, our approach aims at detecting the device with only one slot transmission.

It is noteworthy that RBSA can be regarded as a variant of the slotted Aloha access protocol. Indeed, RBSA does not exclude the possibility of benefiting from the features of other access schemes, such as spreading or repetition. This observation highlights that there is room for improvement in terms of efficiency and robustness to time and frequency offsets. In this work, we do not intend to conduct a thorough comparison with existing access, but to show that RBSA builds a strong foundation to harness spatial diversity gains in the random access channel. We see the access schemes that do not exploit the spatial dimension, as complementary solutions to assist RBSA in detecting packets. However, enabling RBSA to use more complex transmission and detection schemes may not be a trivial task. Since RBSA exhibits remarkable features that are not sufficiently studied, in this paper we study RBSA in its basic form. This means that we follow the assumption of time synchronization. Allowing RBSA to relax synchronization requirements will reduce the complexity of the access scheme at transmission. The required modifications to make RBSA asynchronous are deferred to future works.

Bearing in mind the good performance achieved by RBSA in terrestrial networks in terms of probability of collision and achievable rate, the work presented in this paper investigates its application to massive LEO satellite communication systems operating at Ka-band for massive access. Ka-band has already become the priority spectrum band for some LEO satellite operators [21], which incorporate leading-edge technologies and features, such as sophisticated phased array antennas on each satellite to create multiple dynamic beams. It is noteworthy to mention that analogously to [16], the work presented in this paper leverages on the massive MIMO technology to achieve shapeable and steerable beams, which is crucial to materializing massive access.

In this paper we depart from the IoT requirements of low-rate services and low-power, and low-cost terminals. We envisage a more futuristic IoT ecosystem, where networks will extend its range to more sophisticated IoT terminals and new segments of IoT services with higher data rate demands. In the same vein, the authors in [22] investigate the application of massive MIMO to high-rate IoT systems. As an example, surveillance and security systems that need to send multiple photos or high-volume sensor data, could benefit from the proposed technology in poor connectivity areas. RBSA could also play a role in maritime IoT to handle large data rates [23]. As for the devices, the application scope should be circumscribed to advanced platforms with tracking antennas, providing the necessary gains at Ka-band. Although RBSA goes beyond the current deployments, it has remarkable features that are suitable for IoT. For instance, RBSA lies within the category of random access schemes that are able to perform blind user detection at the receiver.

A recent study on direct NGEO satellite access over millimeter waves (mmWaves) has conducted a feasibility analysis from a regulatory, UE characteristics, space segment, link budget, and system point of view [24]. Despite the advancements in this new landscape, further investigations need to be carried out to deepen into the role of NGEO satellites in the RAN. For example, to allow spectral coexistence between satellite and terrestrial systems and to improve beam management mechanisms. The intention of this work is to contribute to the development of a new access scheme suitable for mMTC in LEO satellite communications systems. Building upon the work presented in [16], we investigated the application of the RBSA scheme to LEO satellite communications with massive MIMO.The link budget feasibility at Ka-band is studied and the necessity of compensating the delay and the Doppler effects to ease the detection is highlighted. The major contributions of this paper are described in the following:

- A thorough analysis has been conducted to show that the proposed method is able to obtain a beamformer in the direction of the target user, without neither acquiring channel state information nor carrying out an exhaustive search through multiple angles;
- The theoretical throughput has been derived. The expressions reveal that the proposed RSBA can benefit from beamforming techniques to lower the collision probability when a large population of terminals is transmitting simultaneously;
- Practical implementation aspects have been tackled, such as the estimation of the covariance matrices and the determination of the number of users;
- We have shown by simulations that the proposed beamforming technique is able to distinguish and separate users that are located in different spots. Numerical results also reveal that performance gains can be achieved with respect to fixed beamforming networks.

The rest of the paper is organized as follows. The application of massive MIMO to LEO satellite communications is discussed in Section 2. In Section 3, we formulate the system model. Section 4 describes the beamforming design and conducts the throughput analysis. The numerical results are presented in Section 5 and finally, the conclusions are drawn in Section 6.

## 2. Massive MIMO in LEO Satellites for Massive IoT

To deal with a high number of communications, satellite systems typically tessellate the coverage area in multiple spotbeams by means of array antenna systems. This paves the way to handling users’ traffic needs, since each beam is processed separately, which scales down the problem. In addition, in order to increase the spectral efficiency, high frequency reuse can be applied. In this case, MIMO precoding techniques must be implemented to handle the interbeam interference. In general, these techniques have been studied in a single feed per beam payload architecture [25]. However, the recent needs of flexible satellite systems, which are capable of accommodating varying capacity demand distributions, raised the interest of the industry in active antenna systems, where many feeds can be combined to synthesize arbitrary beam shapes. This interest is growing in parallel with the development of massive MIMO [26] technology at terrestrial wireless base stations in 5G.

Motivated by the latest developments in 5G technologies, some works have proposed approaches to massive MIMO for satellite communications [27], where the key challenges to adopt massive MIMO in satellites are studied. The work in [28] further works in this direction and exploits massive MIMO when a large-scale active phased array antenna system is equipped at the LEO satellite side. We propose to further work in this direction and design adaptive beamformers for direct radiating arrays. This solution seems to adapt very well to the necessity of pointing specific areas by employing narrow beams, and thus, increase the directivity of the antennas and compensate the higher propagation losses in satellite mm-wave communications at Ka-band. Otherwise, note that, for satellites in LEO, the orbital dynamics make the adoption of conventional satellite precoding techniques (i.e., with fixed beams) very challenging.

**Figure 1:**
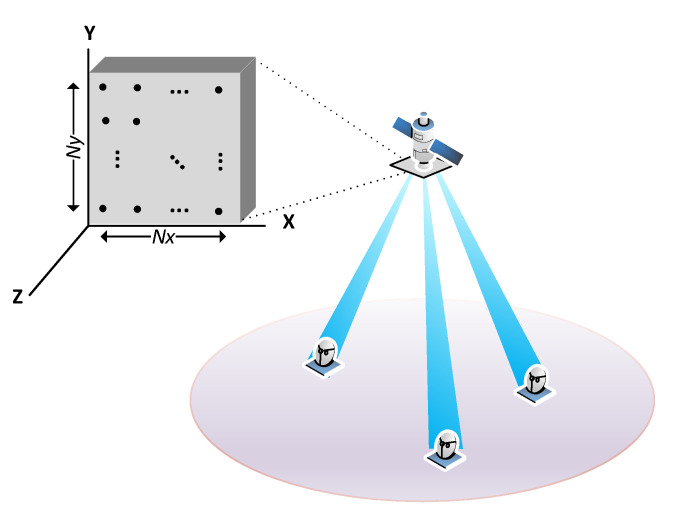
Illustration of a satellite communication system based on narrow digital beams.

The present work studies the practicality of a smart beamforming technique to enable IoT access to a LEO satellite that is equipped with a digital beamforming phased array. The proposed setup departs from the hybrid beamforming architecture, where there is a fixed beamforming matrix. The proposed spatial processing provides the needed flexibility to steer the LEO reception pattern to any designed location. The idea is illustrated in Figure 1. The main feature of the proposed beamformer is its blind nature; thus, it does not need the acquiring channel state information at the receiver (CSIR) nor does it need to make a beam scan in the spatial domain. Hence, it is also an open-loop beamforming technique. In this way it overcomes the cumbersome implementation of precoding schemes requiring user feedback due to the adopted frequency division duplexing (FDD) in satellite communications. Furthermore, the proposed beamformer does not require any previous channel estimation, making it very suitable for IoT grant-free (GF) access, and bypassing the impossibility to use time division duplexing (TDD) schemes in satellite systems [2]. In addition, the satellite line of sight channel simplifies the problem of direction of arrival user identification, which is intricate in terrestrial communications due to the ambiguity that multipath introduces. Finally, the implementation of the proposed smart beamforming, as directly weighting the output of each active antenna element, will allow the system to reduce the collision probability in massive IoT access.

## 3. System Model

Current wireless systems are not designed to support massive connectivity with a large number of devices. The conventional random access consists of four steps: (i) Random access preamble transmission, (ii) random access response, (iii) connection request message, and (iv) contention resolution phase. Then, resources are allocated to users so that subsequent communications occur on scheduled channels. However, in the context of massive connectivity, this procedure is inefficient. The handshake required in the access procedure to allocate resources to users involves a close-loop signaling and multiple exchange of messages. To improve the spectrum efficiency, the use of bulky procedures to establish the link should be avoided. Hence, typical assumptions are not applicable to satellite massive IoT scenarios. To overcome the issues related to the traditional 4-step procedure, we propose the adoption of GF access schemes. GF access is characterized to allow devices to transmit data without waiting for a grant. Therefore, the overhead and latency are reduced. However, the complexity is placed at the receive side, as the detector must be able to decode the data when users transmit on shared time and frequency resources. At the user side, the idea is to combine a reference sequence with the data into a single message. With this configuration, the format of the reference sequence will dictate the strategy followed by the receiver to avoid possible collisions.

The proposed RSBA is based on GF access and its goal is to reduce the probability of collision by combining S-NOMA and spatial signal processing. For the scenario under study, the S-NOMA makes use of the repetition division multiple access (RDMA) to ease the beamforming design. This signature-based scheme employs different repetition patterns at the sample level to design device-specific signatures, providing time diversity. This feature is exploited by the detector that we are proposing so as to create blind beamformers pointing at the user of interest, thanks to the redundancy that RDMA presents. It is important to remark that RBSA is a GF access scheme that lies within the category of Aloha access protocols. Therefore, the proposed scheme is well suited for IoT scenarios where the terminals transmit without requesting permission from the base station. To formulate the system model, let $x_{k}\left[n\right]$ be the baseband signal transmitted by the *k*-th terminal, namely,

(1) $$x_{k}\left[n\right]=\sum_{m=0}^{N_{S}-1}x_{k}^{m}\left[n-Nm\right],$$

for $k=1,\cdots ,N_{U}$. Note that the transmitted signal relies on a packet format to transmit $N_{S}$ symbols consecutively in a time division multiplexing fashion and with a symbol spacing of *N* samples. We assume that the symbols span *M* samples, i.e., $x_{k}^{m}\left[n\right]\neq 0$, for n∈{0,⋯,M−1} and 0 otherwise. This is a sufficiently general model to represent different multicarrier schemes. We favor multicarrier in lieu of single-carrier to achieve a high degree of commonality with 5G. It must be emphasized that users are not frequency multiplexed, but they occupy the whole bandwidth. Interestingly, for N=M, the notation can be used to model orthogonal frequency division multiplexing (OFDM) and single carrier frequency division multiplexing (SC-FDM) waveforms. For M>N, overlapping is allowed. This case lies within the category of filter bank multicarrier (FBMC). To achieve a high user terminal power efficiency, which is of paramount importance to achieve long transmission ranges, it is desirable to avoid signals that exhibit peaks or sudden drops. In conclusion, low peak to average power ratio (PAPR) is a desirable feature. Special attention must be paid to discrete Fourier transform (DFT) spreading techniques to create single-carrier like signals [29,30], which are natively more robust to non-linear effects.

As the waveform design is beyond the scope of the paper, we will adhere to the simple case where N=M, which embraces OFDM-like waveforms. To describe the RDMA scheme, we focus the attention on a given symbol. To ease the analytical tractability we use the following matrix notation $x_{k}^{m}=\left[x_{k}^{m}\left[0\right],\cdots ,x_{k}^{m}\left[N-1\right]\right]$. The approach that is followed to create the signature is based on introducing redundant samples, which are a repetition of some specific samples. This operation can be done by applying a linear processing that results in a new sequence, i.e.,

(2) $${\bar{x}}_{k}^{m}=x_{k}^{m}G=x_{k}^{m}\left[I_{N}\;\;C_{p_{k}}\right]=\left[x_{k}^{m}\;\;x_{k}^{m}C_{p_{k}}\right]=\left[{\bar{x}}_{k}^{m}\left[0\right],\cdots ,{\bar{x}}_{k}^{m}\left[N+L_{R}-1\right]\right]$$

(3) $$C_{p_{k}}={\left[0_{\left(p_{k}-1\right)L_{R}\times L_{R}}^{T}\;\;I_{L_{R}}\;\;0_{N-p_{k}L_{R}\times L_{R}}^{T}\right]}^{T},$$

for $p_{k}\in \left\{1,\cdots ,P\right\}$. Essentially, at the end of the sequence we append a subset of samples of length $L_{R}$. Hence, the original signal is preserved. Now, the duration of the sequences is increased, resulting in:

(4) $${\bar{x}}_{k}\left[n\right]=\sum_{m=0}^{N_{S}-1}{\bar{x}}_{k}^{m}\left[n-(N+L_{R})m\right].$$

The average energy per sample conforms to:

(5) $$P_{T}=\frac{1}{N+L_{R}}E\left\{\left|\right|{\bar{x}}_{k}^{m}{\left|\right|}^{2}\right\}.$$

**Figure 2:**
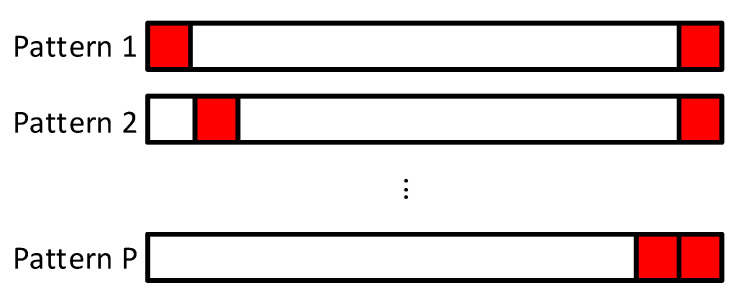
Sequence structure for the RSBA scheme.

Notice that by repeating different parts of the sequence we can create different patterns. This feature can be exploited by the receiver to distinguish users, as long as different repetition patterns are used. This is equivalent to selecting different $C_{p_{k}}$ matrices, as $\left\{C_{1},\cdots ,C_{P}\right\}$ form an orthogonal basis. It is important to remark that the length of the sequence *N* and the length of the repeated part $L_{R}$ will determine the number of different repetition schemes. The idea is illustrated in Figure 2.

Before providing the details of the detection scheme, we formulate the system model. In LEO satellite communication systems at Ka-band, the terminal must establish directional transmission links to compensate for the high path loss experienced at the higher frequencies and to sustain an acceptable link quality. If the terminal is equipped with a global navigation satellite system (GNSS) receiver and is provisioned with the satellite ephemeris, then it can geometrically select the closest satellite and steer a beam towards it. At the other end of the link, the satellite receives multiple signals that come from different areas. In the uplink transmission, the purpose of the satellite is to harness on-board processing to discriminate users by designing directional beamformers. To this end, the satellite is equipped with a planar array of $N_{R}$ elements, which are identical. The separation between adjacent antennas is denoted *d*. For isotropic radiating elements and d=λ/2, the 3-dB beamwidth of a planar array is $\theta_{B}=\frac{100}{\sqrt{N_{R}}}$ [31]. Stacking column-wise the output of each antenna, the received signal becomes,

(6) $$y\left[n\right]=\sum_{k=1}^{N_{U}}h_{k}e^{j2\pi \epsilon_{k}n}{\bar{x}}_{k}\left[n-\tau_{k}\right]+w\left[n\right],$$

where $h_{k}\in C^{N_{R}\times 1}$, $\tau_{k}\in N$, and $\epsilon_{k}\in R$ correspond to the channel vector, the delay, and the carrier frequency offset, respectively, associated to the *k*-th user. Notice that multiple signals are received on shared time and frequency resources. The carrier frequency offset encompasses oscillator uncertainties and the uncompensated Doppler shift that results from the orbital motion, which can be characterized with the analytical expressions provided in [32]. In (6), we have assumed that the frequency misalignment produces a constant rotation during the packet transmission. Note that the reception is contaminated by the noise vector $w\left[n\right]\in C^{N_{R}\times 1}$. To model the channel we have assumed that terminals are in LoS conditions. Hence, upon neglecting the multipath effect, we end up with:

(7) $$h_{k}=\sqrt{\frac{G_{k}G_{R}\left(\theta_{k}\right)}{L_{k}K_{B}TB_{W}}}s_{k}$$

(8) $${\left[s_{k}\right]}_{n+N_{x}m}=e^{j\frac{2\pi d}{\lambda }\left(n\sin\left(\theta_{k}\right)\cos\left(\phi_{k}\right)+m\sin\left(\theta_{k}\right)\sin\left(\phi_{k}\right)\right)}=e^{j\frac{2\pi d}{\lambda }\left(nu_{k}+mv_{k}\right)},$$

for $0\leq n\leq N_{x}-1$ and $0\leq m\leq N_{y}-1$. Thus, $N_{x}N_{y}=N_{R}$. We use ${\left[a\right]}_{i}$ to refer to the *i*-th element of the vector a. The phase term is formulated as a function of the carrier wavelength λ and the direction of the *k*-th signal that is located at elevation angle $\theta_{k}$ and azimuth angle $\phi_{k}$. The angles are measured with respect to the satellite antenna boresight direction. Let $u_{k}=\sin\left(\theta_{k}\right)\cos\left(\phi_{k}\right)$ and $v_{k}=\sin\left(\theta_{k}\right)\sin\left(\phi_{k}\right)$ denote the (u,v)-coordinates of the *k*-th user. The magnitude of the channel depends on the antenna gain of the *k*-th terminal $G_{k}$, the antenna gain of the radiating element $G_{R}\left(\theta_{k}\right)$, the free space loss $L_{k}$, the Boltzmann constant $K_{B}$, the receiver noise temperature *T*, and the carrier bandwidth $B_{W}$. The radiation pattern of antenna elements can be approximated by $G_{R}\left(\theta_{k}\right)\approx \sqrt{G_{R}}\cos^{q}\left(\theta_{k}\right)$, with no azimuth dependence [33]. Furthermore, the *q*-factor can be formulated as a function of the gain as $q=\frac{1}{4}G_{R}-\frac{1}{2}$ [27]. The propagation losses are defined in dB as:

(9) $$L_{k}=32.45+20\log_{10}\left(f_{c}\right)+10\log_{10}\left(d_{k}\right),$$

where $f_{c}$ is the carrier frequency in GHz and $d_{k}$ denotes the slant range in meters along the direction of the *k*-th user. According to [27], we can relate the slant range to the off-nadir angle $\theta_{k}$ as follows:

(10) $$\begin{array}{cc} d_{k}= & \left[R_{E}^{2}+{\left(R_{E}+h\right)}^{2}-2R_{E}\left(R_{E}+h\right)\sin\left(\cos^{-1}\left(\frac{R_{E}+h}{R_{E}}\sin\theta_{k}\right)\right)\right.\\ & {\left.+\sin^{-1}\left(\frac{R_{E}}{R_{E}+h}\right)\left(\frac{R_{E}+h}{R_{E}}\sin\theta_{k}\right)\right]}^{1/2}, \end{array}$$

where *h* is the altitude of the satellite and $R_{E}$ is the Earth’s radius. As [4] shows, the slant range can also be expressed in terms of the elevation angle of the terminal $\psi_{k}$, namely,

(11) $$\begin{array}{cc} d_{k}= & R_{E}\left[\sqrt{{\left(\frac{h+R_{E}}{R_{E}}\right)}^{2}-\cos^{2}\psi_{k}}-\sin\psi_{k}\right]. \end{array}$$

**Figure 3:**
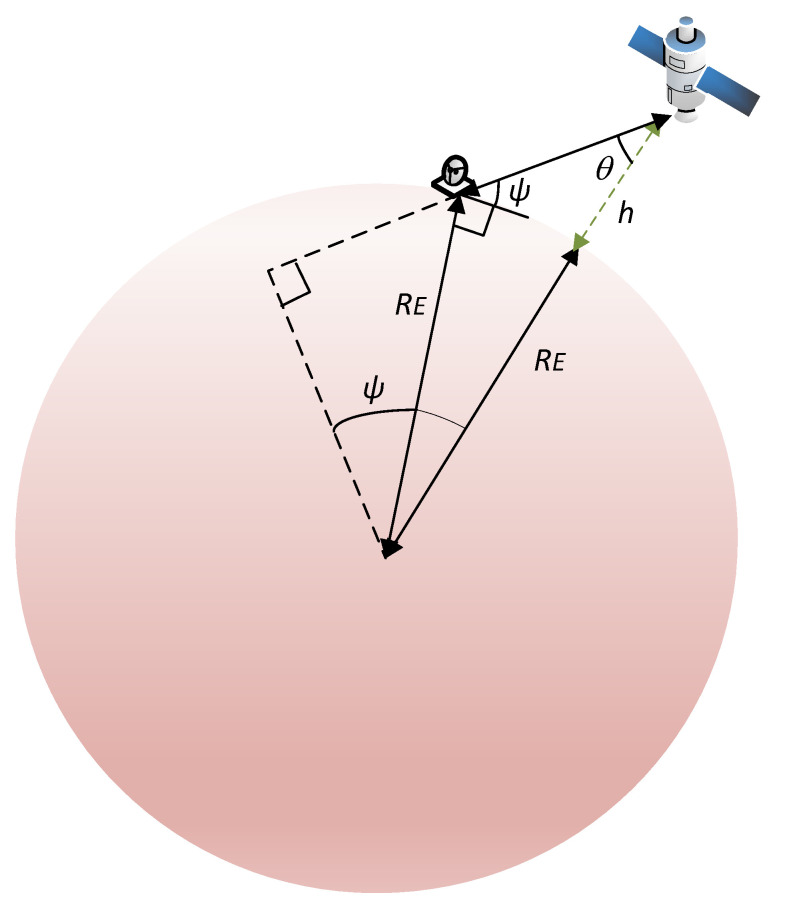
Satellite geometry.

In Figure 3 we have depicted the satellite geometry to have a more clear understanding of the expressions. Notice that $\psi_{k}$ is measured in the horizon plane.

### 3.1. Compensation Strategies

The input-output relation formulated in (6) models an asynchronous GF access scheme, where signals arrive at different time instants. However, by considering more advanced terminals, different levels of synchronization can be achieved. If the terminal is able to detect downlink synchronization signals and extract the system information, the misalignment can be reduced to a high extent. Upon acquiring the system information, the terminal should be able to calculate the delay and the Doppler shift of a given reference point. These values can be used to adopt the time of reference of the satellite. The idea is to compensate time and frequency offsets associated to the reference point in the uplink transmission. Unfortunately, the terminal is unlikely to be located on the same spot that the reference point. Hence, at the receiver, residual time and frequency errors should be expected when terminals are not near the reference point. It becomes evident that the higher the coverage area, the higher will be the residual errors. When strict synchronization cannot be attained, the receiver should be able to find the beginning of the sequence by resorting to the sliding window mechanism [13]. This highlights the necessity of embedding a random access signal within the packet. In a more advantageous situation, when the terminal is aware of the satellite’s trajectory and has localization information, the delay and the Doppler shift can be compensated at the transmit side with high accuracy. Then, signals transmitted from different terminals are time-aligned when reaching the satellite. In such a case, (6) can be particularized for $\tau_{k}=\epsilon_{k}=0$, ∀k. It is worth highlighting that localization information can be obtained even if the GNSS service is not available [34].

### 3.2. Link Budget

In order to assess the feasibility of a direct LEO satellite access at Ka-band, the link budget analysis must be conducted. The system parameters are gathered in Table 1. It is noteworthy to mention that the effective isotropic radiated power density (EIRPD) and the antenna gain-to-noise-temperature have been obtained from [35]. Remarkably, based on the link budget we can compute the signal-to-noise-ratio (SNR) in dB, which indicates the reliability of the link, as:

(12) $$\mathrm{SNR}=EIRPD+G/T-K_{B}-32.45-20\log_{10}\left(fc\right)-10\log_{10}\left(d_{\mathrm{MAX}}\right).$$

The term 32.45 corresponds to $20\log_{10}\left(4\pi \times 10^{9}/3\times 10^{8}\right)$. The model can be further characterized with the atmospheric loss, the scintillation loss, the shadowing margin, and the co-channel interference [35]. It is important to remark that for array antenna systems, the antenna gain-to-noise-temperature becomes:

(13) $$G/T=G_{R}+10\log_{10}\left(N_{x}N_{y}\right)-10\log_{10}\left(T\right),$$

where *T* denotes the system temperature. Particularizing for the values of Table 1, we get SNR=16.2 dB. It must be highlighted that this value cannot be attained with low-cost and low-power terminals at the Ka-Band; thus, this paper focuses on more powerful broadband IoT terminals.

The link budget analysis could be further complemented by including signal characteristics, such as the data rate, the modulation and coding scheme, and the bit rate. To provide the additional information we need to determine the range of received SNRs, the possible carrier-to-interference ratio (C/I) levels, the block length, and the required energy-bit-to-noise ratio (Eb/N0), to mention a few. To this end, we would need to conduct a more thorough analysis. Otherwise, it is difficult to make a judicious selection and determine the most suitable signal characteristics. It is worth highlighting that the paper is essentially focused on the design of a blind and open-loop beamforming technique in the context of satellite communications with massive MIMO. Since the physical layer design is not a trivial task and it is not within the main scope of the paper, we defer it to future work.

## 4. Resource Sharing Beamforming Access

For ease exposition, the detection scheme will be described under the premise that users are time and frequency synchronized, i.e., $\epsilon_{k}=\tau_{k}=0$, ∀k. Hence, RBSA in its basic form is based on slotted Aloha. It is also important to mention that a peculiarity of slotted Aloha access schemes is that the terminal EIRP could be dictated by the aggregate throughput of the system rather than the terminal throughput [13]. This drawback, which is inherent to systems where the terminal has a limited time to transmit the packet with respect to the frame duration, may increase the cost of the device. Interestingly, unslotted random access schemes based on either frequency division multiple access (FDMA) or code division multiple access (CDMA) are more suitable for low-cost terminal solutions. In this section we will show that under time slot synchronization, the receiver is able to spatially separate users via smart beamforming. Thanks to the repetition scheme introduced in Section 3 we can provide spatial diversity multiple access as described next. First, we gather the samples as follows $Y_{m}=\left[y\left[m\left(N+L_{R}\right)\right],\cdots ,y\left[\left(m+1\right)\left(N+L_{R}\right)-1\right]\right]$, for $m=0,\cdots ,N_{S}-1$. Essentially, we group the samples to operate on a symbol basis. With this arrangement, we can get:

(14) $$Y_{m}=\sum_{k=1}^{N_{U}}h_{k}{\bar{x}}_{k}^{m}+W_{m},$$

where $W_{m}=\left[w\left[m\left(N+L_{R}\right)\right],\cdots ,w\left[\left(m+1\right)\left(N+L_{R}\right)-1\right]\right]$. The system is modeled like a $N_{R}\times \left(N+L_{R}\right)$ MIMO communication scheme. For simplicity we will omit the symbol index from here onwards. The data set can be split into the data and the redundant samples, yielding,

(15) $$Y=\left[Y_{d}\;\;Y_{r}\right]=\sum_{k=1}^{N_{U}}h_{k}\left[x_{k}\;\;x_{k}C_{p_{k}}\right]+\left[W_{d}\;\;W_{r}\right].$$

**Figure 4:**
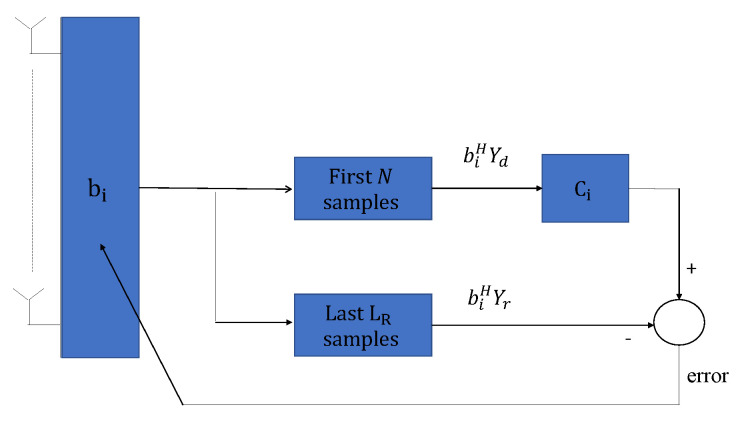
Scheme of the beamformer design at base band.

At the satellite, a beamvector $b_{i}\in C^{N_{R}\times 1}$ is designed such that it presents a maximum at the direction of arrival (DoA), where the signal with repetition pattern $C_{i}$ comes from. The constrained optimization problem is posed as:

(16) $$\begin{array}{cc} \min_{b_{i}} & E\left\{\left|\right|b_{i}^{H}\left(Y_{d}C_{i}-Y_{r}\right){\left|\right|}^{2}\right\}\\ s.t. & E\left\{b_{i}^{H}Y_{d}C_{i}Y_{r}^{H}b_{i}+b_{i}^{H}Y_{r}C_{i}^{H}Y_{d}^{H}b_{i}\right\}=\rho , \end{array}$$

where ρ is some constant different from zero. Figure 4 sketches the proposed design. Under the assumption that signals from different users are statistically independent and that the noise is uncorrelated with the data, we can formulate the mean square error as:

(17) $$E\left\{\left|\right|b_{i}^{H}\left(Y_{d}C_{i}-Y_{r}\right){\left|\right|}^{2}\right\}=b_{i}^{H}\left(R_{i}+R_{r}\right)b_{i}-b_{i}^{H}\left(R_{ir}+R_{ir}^{H}\right)b_{i}$$

(18) $$R_{i}=L_{R}I_{N_{R}}+\sum_{k=1}^{N_{U}}E\left\{\left|\right|x_{k}C_{i}{\left|\right|}^{2}\right\}h_{k}h_{k}^{H}$$

(19) $$R_{r}=L_{R}I_{N_{R}}+\sum_{k=1}^{N_{U}}E\left\{\left|\right|x_{k}C_{p_{k}}{\left|\right|}^{2}\right\}h_{k}h_{k}^{H}$$

(20) $$R_{ir}=\sum_{k=1}^{N_{U}}E\left\{x_{k}C_{i}C_{p_{k}}^{H}x_{k}^{H}\right\}h_{k}h_{k}^{H}.$$

To get the matrices, we rely on the fact that the noise samples are independent and identically distributed with $E\left\{WW^{H}\right\}=\left(N+L_{R}\right)I_{N_{T}}$. In alignment with (7), the noise samples have unit variance. The optimal beamformer can be computed by setting the partial derivatives of the Lagrangian to zero, thus obtaining:

(21) $$\left(R_{i}+R_{r}\right)b_{i}=\left(1+\lambda \right)\left(R_{ir}+R_{ir}^{H}\right)b_{i},$$

where λ is the Lagrange multiplier. The solution consists in computing the eigenvalue decomposition of matrices $\left(R_{i}+R_{r}\right)\in C^{N_{R}\times N_{R}}$ and $\left(R_{ir}+R_{ir}^{H}\right)\in C^{N_{R}\times N_{R}}$. It can be verified that the mean square error is minimum for minimum λ. Therefore, the optimal $b_{i}$ is selected as the eigenvector associated to the eigenvalue that is closest to 1. Another important results is that the minimum variance (MV) beamformer, namely:

(22) $$b_{i}=\frac{R_{i}^{-1}h_{d}}{h_{d}^{H}R_{i}^{-1}h_{d}},$$

is solution of (21), as long as the rank of $R_{ir}$ is one. This is tantamount to saying that there is just one user transmitting with the pattern $C_{i}$. Without loss of generality, the array vector in the direction of the desired user is denoted $h_{d}$, where $d\in \left\{1,\cdots ,N_{U}\right\}$. Accordingly, the signal-to-interference-plus-noise ratio (SINR) can be formulated as:

(23) $${\mathrm{SIN}R}_{i}=\frac{|b_{i}^{H}h_{d}{|}^{2}}{\sum_{k\neq d}{\left|b_{i}^{H}h_{k}\right|}^{2}+\frac{\left|\right|b_{i}{\left|\right|}^{2}}{P_{T}}}.$$

To create the advantageous situation where the rank of $R_{ir}$ is one, $P\geq N_{U}$ must be satisfied. Thus, for a given $N_{U}$, the performance is enhanced by increasing the number of repetition patterns *P*. It becomes evident that if $P>>N_{U}$, it is highly unlikely that two users transmit with the same repetition structure. In exchange, the complexity is increased, as it scales with *P*.

The procedure can be extended to the situation where multiple terminals pick the same repetition pattern. Let $N_{U_{i}}$ denote the number of terminals that employ $C_{i}$ to obtain the redundant symbols. Assuming that the number of active terminals $N_{U_{i}}$ is correctly estimated, which will be tackled later on, the satellite must perform a specific signal processing for each terminal. As Appendix A proves, for large scale antenna arrays, this boils down to selecting the $N_{U_{i}}$ eigenvectors associated to the $N_{U_{i}}$ eigenvalues that are closest to 1. Each of them tends to the MV beamformer (22) in the direction of the terminal that is targeted. The beauty of the proposed beamforming technique is that the terminals can be distinguished and separated, as long as they are not located in the same spot, without estimating the channel nor making a beam scan.

In practical situations, $R_{i}$, $R_{r}$, and $R_{ir}$ must be estimated by their corresponding sample-covariance matrices. This entails a complexity increase with respect to the fixed grid beamforming network, which is a simpler solution. However, the numerical results presented in Section 5 show that the computational complexity translates into a significant SINR increase with respect to the fixed beamforming approach. Sticking to the system model formulated in (14), the estimated covariance matrices can be computed as:

(24) $${\hat{R}}_{i}=\frac{1}{N_{S}}\sum_{m=0}^{N_{S}-1}Y_{m,1}C_{i}C_{i}^{H}Y_{m,1}^{H}$$

(25) $${\hat{R}}_{r}=\frac{1}{N_{S}}\sum_{m=0}^{N_{S}-1}Y_{m,2}Y_{m,2}^{H}$$

(26) $${\hat{R}}_{ir}=\frac{1}{N_{S}}\sum_{m=0}^{N_{S}-1}Y_{m,1}C_{i}Y_{m,2}^{H},$$

from the observations:

(27) $$Y_{m,1}=\left[y\left[m(N+L_{R})\right],\cdots ,y\left[m(N+L_{R})+N-1\right]\right]\in C^{N_{R}\times N}$$

(28) $$Y_{m,2}=\left[y\left[m(N+L_{R})+N\right],\cdots ,y\left[\left(m+1\right)\left(N+L_{R}\right)-1\right]\right]\in C^{N_{R}\times L_{R}}.$$

**Figure 5:**
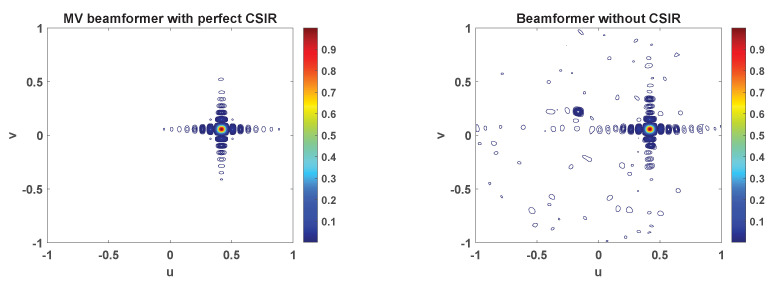
Beampattern displayed in the uv-plane. The beamformer points at a terminal that is located at $\left(\theta ,\phi )=(24.91{}^{\circ },7.89{}^{\circ }\right)$ or, equivalently u=0.4172,v=0.0578. There are nine interfering users and three of these select the same pattern as the user of interest.

To corroborate that the proposed beamforming technique allows to increase the number of terminals without collision, which is the key point of the paper, we have evaluated the beamformer response in different azimuth and elevation angles. We have defined the beampattern as $G\left(\theta_{d},\phi_{d}\right)={\left|b_{i}^{H}s_{d}\right|}^{2}$, for $0\leq \theta_{d}\leq 90{}^{\circ }$ and $0\leq \phi_{d}\leq 360{}^{\circ }$. In Figure 5 we have depicted the beampattern, for $N_{U}=10$, d=λ/2, and $N_{x}=N_{y}=32$. In particular, we have represented the MV beamformer with perfect CSIR and the proposed blind beamformer. For the sake of clarity, the highest value of the beampattern is normalized to 1. Regarding the format of the signal, the terminals employ the OFDM modulation. The frame consists of M=64 subcarriers, which only 58 are active, and $N_{S}=400$ OFDM symbols. Information is conveyed in QPSK symbols. To create the repetition pattern, users can pick any of the P=4 orthogonal signatures. Hence, the redundancy spans $L_{R}=16$ samples. The rest of the system parameters are taken from Table 1. In the case represented in Figure 5, the user of interest is located at $\left(\theta ,\phi )=(24.91{}^{\circ },7.89{}^{\circ }\right)$ or, equivalently u=0.4172,v=0.0578. It is worth underlining that 3 out of the 9 interfering users select the same repetition pattern. In Figure 5 we only plot the first eigenvector, which corresponds to the beamformer that points to the terminal of interest in this simulation. Graphically comparing both schemes, we can resolve that the proposed beamformer technique provides a reasonably good estimate of the MV beamformer.

### 4.1. Determination of the Number of Terminals

The proposed beamforming technique must be aware of the number of terminals that pick a given repetition pattern. To acquire this information one option is to exploit the structure of $R_{ir}+R_{ir}^{H}$, for 1≤i≤P. The analysis conducted in this section reveals that $R_{ir}+R_{ir}^{H}$ is of rank $N_{U_{i}}$. Admittedly, $R_{ir}$ is only available through (26) that is a noisy estimate, owing to the fact that the average is taken over a finite number of samples. Hence, the smallest $N_{R}-N_{U_{i}}$ singular values of ${\hat{R}}_{ir}+{\hat{R}}_{ir}^{H}$ are not equal to zero. Yet, the number of terminals can be estimated in the presence of noise within the framework of signal detection [36]. To benefit from [36], the data must follow a Gaussian model and in addition, an estimate of the autocovariance matrix should be available. Regrettably, the structure of $R_{ir}+R_{ir}^{H}$ is not consistent with an autocovariance matrix. In addition, the data set that is used to estimate (26), which is given by $\left\{Y_{m,1}C_{i}Y_{m,2}^{H}+Y_{m,2}^{H}C_{i}^{H}Y_{m,1}^{H}\right\}$, cannot be modeled as Gaussian signals. Thus, the adoption of detection methods based on the assumption of Gaussian data, such as [36], will yield an uncertain result. To avoid departing from Gaussianity, we refrain from processing $R_{ir}+R_{ir}^{H}$. Alternatively, we consider a different approach to determine the multiplicity of the noise singular values.

The difficulty in estimating $N_{U_{i}}$ from ${\hat{R}}_{ir}+{\hat{R}}_{ir}^{H}$, leads to separately estimate $N_{U}$ and $N_{U}-N_{U_{i}}$. With these two estimates it is straightforward to get $N_{U_{i}}$. First, we focus the attention on $N_{U}$. In light of the findings of this section, we know that $R_{r}$ is a full rank matrix that can be split into signal and noise subspaces. From the singular value decomposition we know that the $N_{U}$ dominant singular values are associated to the signal subspace. The rest of singular values correspond to noise and have the same magnitude. Let $\left\{\gamma_{1},\cdots ,\gamma_{N_{R}}\right\}$ be the sample singular values of ${\hat{R}}_{r}$. In light of Appendix A discussion, we can assume that in OFDM-like signals, $y\left[j\right]$ and $y\left[l\right]$ are statistically independent, for j≠l. Therefore, the column vectors that form $\left\{Y_{m,2}\right\}$ can be assumed to be statistically independent and can be modeled as Gaussian random vectors. These properties allow us to benefit from the theory developed in [36] to estimate the number of signals as:

(29) $${\hat{N}}_{U}=\underset{0\leq l\leq N_{R}-1}{\mathrm{argmin}}{\mathrm{AIC}}_{\gamma }\left(l\right),$$

(30) $${\mathrm{AIC}}_{\gamma }\left(l\right)=-2\left(N_{S}L_{R}\right)\log\left[\frac{\prod_{i=l+1}^{N_{R}}\gamma_{i}}{{\left[\frac{1}{N_{R}-l}\sum_{i=l+1}^{N_{R}}\gamma_{i}\right]}^{N_{R}-l}}\right]+2l\left(2N_{R}-l\right).$$

Next, we tackle the estimation of $N_{U}-N_{U_{i}}$. Note that if we subtract the redundant samples $Y_{m,2}$ from $Y_{m,1}C_{i}$, we get:

(31) $$Z_{m,i}=Y_{m,1}C_{i}-Y_{m,2}=\sum_{k\in N_{i}^{c}}h_{k}\left(x_{k}C_{i}-x_{k}C_{p_{k}}\right)+W_{m,1}C_{i}-W_{m,2},$$

where $N_{i}^{c}$ is the complementary set of $N_{i}$. This means that $N_{i}^{c}$ gathers the index of those users that do not use the pattern $C_{i}$. The noise matrices are defined as:

(32) $$W_{m,1}=\left[w\left[m(N+L_{R})\right],\cdots ,w\left[m(N+L_{R})+N-1\right]\right]\in C^{N_{R}\times N}$$

(33) $$W_{m,2}=\left[w\left[m(N+L_{R})+N\right],\cdots ,w\left[\left(m+1\right)\left(N+L_{R}\right)-1\right]\right]\in C^{N_{R}\times L_{R}}.$$

The remarkable property of (31) is that the signal subspace of $Z_{m,i}$ has dimension $N_{U_{i}}^{c}=N_{U}-N_{U_{i}}$. In addition, the singular values of the noise subspace have the same magnitude. As a result, analogously to the procedure that is followed to estimate $N_{U}$, we can leverage on the sample-covariance matrix of $Z_{m,i}$ to determine $N_{U_{i}}^{c}$. Bearing in mind that the column vectors of $Z_{m,i}$ are statistically independent and that they obey the Gaussian distribution, we can obtain the second estimate as:

(34) $${\hat{N}}_{U_{i}}^{c}=\underset{0\leq l\leq N_{R}-1}{\mathrm{argmin}}{\mathrm{AIC}}_{\lambda }\left(l\right),$$

(35) $${\mathrm{AIC}}_{\lambda }\left(l\right)=-2\left(N_{S}L_{R}\right)\log\left[\frac{\prod_{i=l+1}^{N_{R}}\lambda_{i}}{{\left[\frac{1}{N_{R}-l}\sum_{i=l+1}^{N_{R}}\lambda_{i}\right]}^{N_{R}-l}}\right]+2l\left(2N_{R}-l\right)$$

where $\left\{\lambda_{1},\cdots ,\lambda_{N_{R}}\right\}$ represent the singular values of:

(36) $${\hat{R}}_{z}=\frac{1}{N_{S}}\sum_{m=0}^{N_{S}-1}Z_{m,i}Z_{m,i}^{H},$$

for 1≤i≤P. It is evidenced from (29) and (34) that the number of users that choose the *i*-th repetition pattern is computed as ${\hat{N}}_{U_{i}}={\hat{N}}_{U}-{\hat{N}}_{U_{i}}^{c}$.

### 4.2. Throughput Analysis

This section is devoted to conduct the throughput analysis of the RSBA scheme. To determine under what conditions the satellite can successfully collect the data, we rely on the analytic model of a time-slotted network. The duration of the time slot is equal to the packet transmission time. First, we focus on the collision probability of a specific terminal. In the scenario under study, a collision occurs when there is overlap in time and spatial domains. If we focus on the case that two users are attempting to send data, the overlap in the spatial domain can be seen in the uv-plane. In alignment with the satellite field of view, the (u,v)-coordinates $\left(u_{k},v_{k}\right)$ must satisfy $u_{k}^{2}+v_{k}^{2}\leq \sin^{2}\left(\theta_{\mathrm{MAX}}\right)$. The minimum elevation angle of the terminal and satellite orbit will determine $\theta_{\mathrm{MAX}}$. We declare that the *i*-th and the *j*-th users collide if $|u_{i}-u_{j}{|}^{2}+{\left|v_{i}-v_{j}\right|}^{2}\leq \Delta_{uv}^{2}$. The parameter $\Delta_{uv}$, which determines the minimum angular separation to successfully separate users, depends on the spatial processing performed at the receiver.

Under the premise that users are uniformly located in the coverage area, the probability that the undesired user is located in the same spot as the desired user is given by $P_{c}=\frac{\pi \Delta_{uv}^{2}}{\pi \sin^{2}\left(\theta_{\mathrm{MAX}}\right)}$. When there is no angular discrimination, $P_{c}=1$. Thus, we can establish that the probability that a packet is successfully received in the presence of *k* interfering users is given by ${\left(1-P_{c}\right)}^{k}$. Following the guidelines reported in [37] and assuming that the arrivals follow the Poisson distribution, it follows that the probability of successful transmission for the target terminal is $P_{S}=e^{-GP_{c}}$, where *G* denotes the average number of transmission attempts by all the users during the packet transmission. Recalling that the throughput is the average number of transmission attempts per packet duration multiplied by the probability of success, the performance metric in packets/slot can be defined as:

(37) $$\eta_{T}\left(P_{S}\right)=GP_{S}=Ge^{-GP_{c}}.$$

Adopting the same approach as [9], the number of users that the network can support for a given probability of successful transmission is approximated to:

(38) $$N_{\max}\approx \frac{\eta_{T}\left(P_{t}\right)}{d_{a}}.$$

In notation terms, $\eta_{T}\left(P_{t}\right)$ correspond to the maximum achievable throughput at $P_{S}=P_{t}$, which is the desired probability of successful transmission. Finally, $0\leq d_{a}\leq 1$ is the average activity of the users. It is important to remark that $N_{\max}$ is significantly higher than $N_{U}$, which corresponds to the active users at a given time slot. It is also worth mentioning that the throughput is computed under the assumption that the satellite can always direct a beam towards each active user. In a nutshell, the analysis assumes that colliding packets can be decoded as long as terminals are sufficiently separated. This is a pessimistic assumption. Indeed, the throughput could be increased by employing more advanced receivers based on iterative successive interference cancellation (SIC). In such a case, the receiver benefits from the capture effect to solve packet collisions from users that are in close proximity [9]. To model the effect of the iterative SIC procedure, it is necessary to generate the performance curves of the selected modulation and coding schemes in additive white Gaussian (AWGN) channels. As this level of detail is not provided, the study of the iterative SIC is a topic of future research.

**Figure 6:**
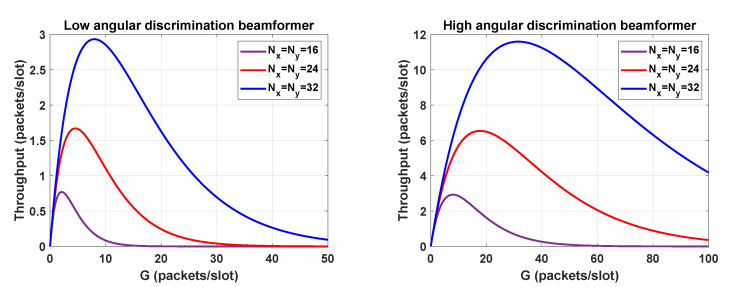
Throughput of RBSA for $\theta_{\mathrm{MAX}}=$ 44.44${}^{\circ }$.

In light of the above discussion, it appears fair to assume that the performance is governed by the array size and the spatial processing. We would expect that the higher the number of antenna elements $N_{x},N_{y}$, the lower the distance $\Delta_{uv}$. In general, for an arbitrary beamformer, it is difficult to formulate $\Delta_{uv}$ in a closed-form expression. As a solution, we may take as reference the response of the phased shift beamformer. For instance, the minimum angular separation could be taken as the 3-dB beamwidth of the phase shift beamformer. As this criterion could be too optimistic, we may alternatively consider the *n*-th null beamwidth (nNB). In the boresight, the nNB is found as $\theta_{nNB}=2\sin^{-1}\left(2n/\sqrt{N_{R}}\right)$, for $N_{x}=N_{y}=\sqrt{N_{R}}$ and $d=\frac{\lambda }{2}$. The relation between (u,v) and (θ,ϕ) leads to $\Delta_{uv}=\sin\left(\theta_{nNB}\right)$. Sticking to the case that $P_{c}=\frac{\sin^{2}\left(\theta_{nNB}\right)}{\sin^{2}\left(\theta_{\mathrm{MAX}}\right)}$ and following the steps described in this section, we have represented the throughput in Figure 6. In particular, we consider a massive MIMO architecture and two beamforming techniques, which achieve different angular discrimination values. The angular resolution of low and high angular discrimination beamformers can be approximated by the 2 NB and the 1 NB, respectively, of the phase shift beamformer. As expected, by increasing the number of antennas and improving the angular resolution, we can increase the load of the network without losing further packets. Note that if the load exceeds a given value, the throughput drops drastically.

## 5. Numerical Results

This section evaluates the GF access procedure described in Section 3 and Section 4 from the link-level perspective. The proposed technique lies within the category of digital beamforming solutions, which refrain from using fixed beamforming matrices. The results presented in this section evidence that although the devised solution entails a complexity increase with respect to the fixed beamforming approach, it achieves a far superior performance. The system parameters are listed in Table 1. The distance between antenna elements is set to d=λ/2. To generate the sequences, we have used the OFDM modulation format. The bandwidth is partitioned into M=64 subcarriers, leaving empty approximately 9% of the subcarriers to reduce out-of-band emissions. Hence, at the edges, three subcarriers remain silent. The symbols have been drawn from the quadrature phase-shift keying (QPSK) constellation. There are P=4 orthogonal patterns of length $L_{R}=16$. To generate the redundant symbols, users pick randomly any of the repetition patterns available. In the following we analyze the impact of several parameters on the performance. Essentially, we focus the attention on the number of symbols per frame $N_{S}$, the number of users $N_{U}$, and the number of antenna elements $N_{x},N_{y}$. In the experimental validation, we have focused the attention on two array antenna configurations, i.e., $N_{x}=N_{y}=24$ and $N_{x}=N_{y}=32$. We assume that users can be located in any spot that is illuminated by the satellite. This means that $\theta_{k}$ and $\phi_{k}$ can take any value within the intervals [0, 44.44${}^{\circ }$] and [0, 360${}^{\circ }$], respectively. Consequently, the free space loss variation is around 3.35 dB.

### 5.1. Number of Terminals

First we evaluate the estimator that is used to determine the number of sources. The accuracy of the method presented in Section 4 is measured as the mean absolute percentage error (MAPE), which is given by:

(39) $$\mathrm{MAPE}=\frac{1}{P}\sum_{i=1}^{P}\left|\frac{N_{U_{i}}-{\hat{N}}_{U_{i}}}{N_{U_{i}}}\right|.$$

**Figure 7:**
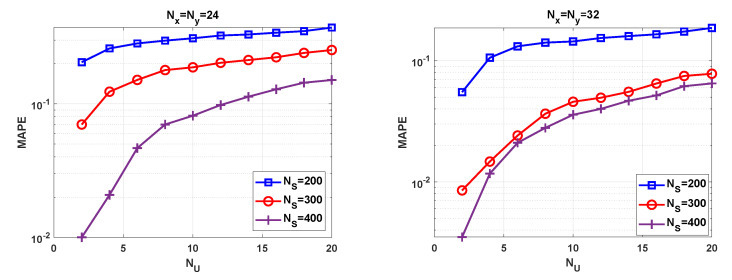
MAPE versus $N_{U}$ for different values of $N_{S}$ and $N_{x},N_{y}$.

In Figure 7, we represent the MAPE against the number of users. As it could be easily anticipated, the higher the number of symbols $N_{S}$, the lower the mismatch error between the sample-covariance and the real covariance matrices. Then, (29) and (34) become more accurate and thus, the MAPE is reduced. To achieve a given accuracy, it is worth highlighting that array antennas consisting of $N_{R}=1024$ elements need less samples than those antennas using $N_{R}=576$ elements. Another interesting conclusion that could be inferred from Figure 7 is that the number of errors committed increases with $N_{U}$. In most of the cases that the test fails to determine the number of terminals, it follows that ${\hat{N}}_{U}<N_{U}$. This implies that the error is due to underestimation and thus, some users are missed.

### 5.2. Signal-to-Interference-Plus-Noise Ratio

**Figure 8:**
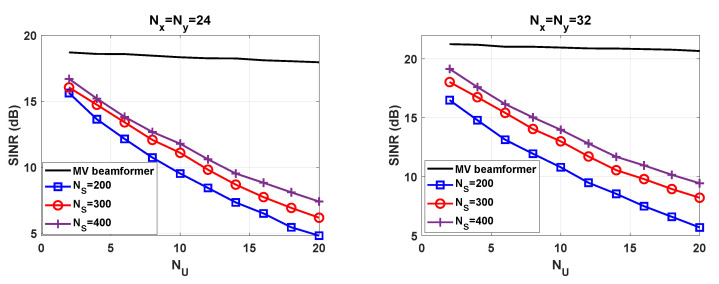
SINR versus $N_{U}$ for different values of $N_{S}$ and $N_{x},N_{y}$.

To complete the experimental validation we evaluate the SINR. With the aim of using the results obtained in Figure 7, we compute the average SINR after applying the proposed beamforming technique in the direction of the detected users. Hence, we evaluate a subset of the $N_{U}$ concurrent users. As a benchmark, we have represented the SINR of the MV beamformer with perfect CSIR. As Figure 8 highlights, the degradation is almost not noticeable when perfect CSIR is available. This confirms that the asymptotic orthogonality exists for large antenna arrays. As the number of users increases, the SINR gap is widened between the beamformer envisaged in this paper and the benchmark. The fundamental reason is that the number of symbols $N_{S}$ and the number of users $N_{U}$ have an impact on the SINR. Having more samples results in more accurate sample-covariance matrices, which is crucial to successfully separating users. As a general statement, high user density scenarios suffer from SINR degradation, unless a sufficiently high number of symbols $N_{S}$ are transmitted. Regarding the antenna configuration, the scheme with $N_{R}=1024$ achieves the best performance. As expected, the array gain and the interference mitigation capability depend on the number of antenna elements.

**Figure 9:**
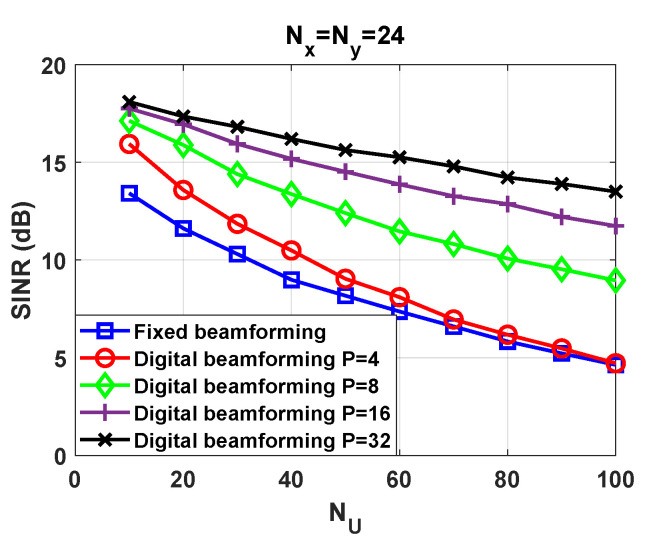
SINR versus $N_{U}$ in fixed and digital beamforming schemes for $N_{x}=N_{y}=24$.

**Figure 10:**
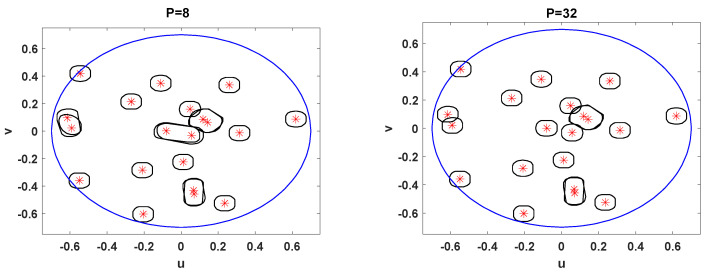
User positions and 4.3 dB contour of the beams generated by the proposed beamforming with a planar array of 24×24 antenna elements.

**Figure 11:**
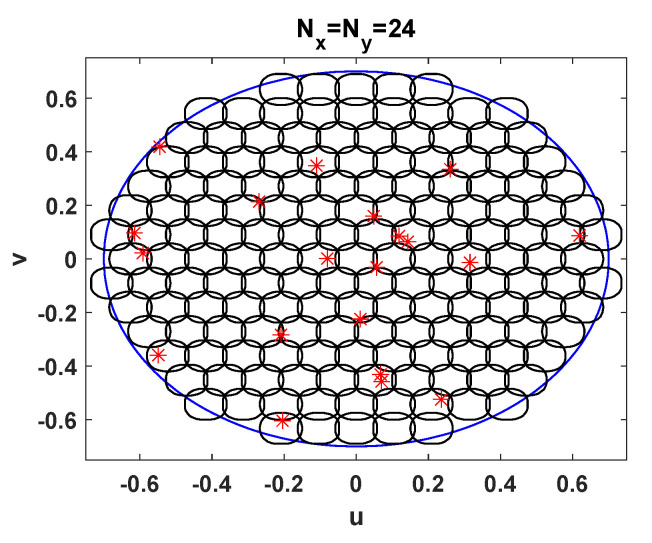
User positions and 4.3 dB contour of the beams generated by a fixed beamforming with a planar array of 24×24 antenna elements.

For M=64 and P=4, the performance loss with $N_{U}$ is remarkable. The results provided in Figure 8 intend to study the impact of $N_{S}$ and $N_{U}$ into the SINR. Unfortunately, the full potential of the beamforming technique is not seen. Towards this end, we carry out an assessment under ideal conditions. That is, we assume that the receiver is able to perfectly estimate the number of active users $N_{U}$ and the covariance matrices formulated in (18)–(20). To gain a better insight we consider different frame configurations. Now, the number of repetition patterns is not kept constant, i.e., $P\in \left\{4,8,16,32\right\}$. The average SINR is depicted in Figure 9. In alignment with the analysis conducted in Section 4, the results reveal that the performance enhances as *P* increases. The slope of the curve is more pronounced with P=4 than in the rest of the cases. To justify the results we include Figure 10 and Figure 11. For P=8,16,32, we have not analyzed the impact of $N_{S}$ on the SINR. The $N_{S}$ value that leads to the best complexity/performance trade-off is left for the future work.

When users are in close proximity and in addition choose the same repetition pattern, a cluster is created. Then, the proposed beamforming technique is not able to mitigate the unwanted signals that come from the cluster. This is clearly seen in Figure 10, where we have represented the beam pattern and the position of users at a given time instant. This situation can be improved to a high extent if nearby users are associated to orthogonal repetition patterns. The probability of this event occurring is increased with *P*. This can be appreciated in Figure 10. Interestingly, when there are P=32 orthogonal repetition patterns, we achieve a finer spatial resolution than in the P=8 counterpart.

The digital beamforming technique described in this work is compared to a fixed beamforming network that divides the coverage area into 161 beams. The beam pattern is represented in Figure 11. The position of users coincides with those of Figure 10. The downside of the fixed beamforming stems from the fact that beams point towards grid points that do not match with user positions. Another adverse effect is that the beamforming network is not designed to mitigate unwanted user positions. For this reason the SINR suffers the highest degradation in Figure 9. To carry out a fair comparison with the digital beamforming, we have assumed ideal conditions as well. As the fixed beamforming network operates with full frequency reuse and adjacent beams are overlapped, the signal of a given user leaks into several beams. Due to the noise and the interference, the system may not choose the closest beam to decode the signal, so that it could be received from the side lobe of a neighboring beam. We have disregarded this situation and thus, the SINR is computed assuming that the signal is extracted from the beam with the highest gain. It is noteworthy to mention that the fixed beams grid approach is evaluated to gain a better insight into the benefits of RBSA, but it is not optimized. The best performance in the context of fixed beamforming will require some radio resource management- (RRM) based assignment to avoid users that are too physically close and are simultaneously transmitting. However, this is not consistent with the GF philosophy and thus, it is not applicable for unscheduled transmissions. Alternatively, the SINR gain could be increased for current terminal offset from the beam center, by having larger beams overlap. The maximization of the SINR in the scenario under study for fixed beamforming matrices is an interesting line of work that will be targeted in the future.

## 6. Conclusions

This paper presents a GF access scheme, which is referred to as RBSA. The proposed scheme is suitable for provisioning service to a large population of terminals that transmit simultaneously without requesting a grant. The RBSA, which was originally conceived for terrestrial communications, has been applied to a LEO satellite communications system with a regenerative satellite payload. It is considered that terminals are able to establish a direct link with the satellite at the Ka-band. In the scenario under study, the satellite is equipped with a uniform planar array that consists of a high number of elements. Hence, the system lies within the category of LEO satellite communications with massive MIMO. The merit of the RBSA is that it is able to exploit the spatial diversity to separate and distinguish users. The idea is to direct narrow digital beams towards the direction of users. The throughput analysis has revealed that the system can leverage on the angular discrimination to reduce the probability of collision, which allows us to increase the load of the network. To fulfil this objective we have designed a blind and open-loop beamformer. Hence, the satellite does not need to estimate the channel, request feedback from terminals, nor make a beam scan in azimuth and elevation. The work presented also takes into account practical implementation aspects, such as the determination of the number of users and the estimation of the covariance matrices. In addition, this work presents new findings about the beamforming design for terminals with the same repetition pattern. Namely, due to the use of a large scale array, these beamformers correspond with different eigenvectors; thus, no direction of arrival estimation is needed in this case to design separate beamformers. Numerical results show that the proposed beamformer design is able to achieve shapeable beams with angular resolution, which allows us to mitigate inter-user interference. As expected, the number of elements has an impact on the array gain and the interference mitigation capabilities. Another important result is that the overall performance depends on the accuracy of the sample-covariance matrices that are used to generate the beamformers. The better the accuracy, the higher the number of users that can access the media without experiencing significant SINR degradation. Therefore, high user density scenarios will require very accurate sample-covariance matrices to decode overlapped signals. To understand the potential of the proposed beamforming technique, we carried out an assessment under ideal conditions, where covariance matrices are perfectly estimated. The most interesting finding is that the higher the number of repetition patterns *P*, the lower the SINR degradation as the number of active users $N_{U}$ increases. The numerical results also evidence that the digital beamforming clearly outperforms a beamforming network that points to a fixed grid.

## Figures and Tables

**Table 1:** System parameters.

Parameters	Value
Carrier frequency	$f_{c}=29$ GHz
Minimum elevation angle	$\psi_{\mathrm{MIN}}=40$ ${}^{\circ }$
Field of view	$\theta_{\mathrm{MAX}}=44.44$ ${}^{\circ }$
Satellite altitude	h=600 Km
Maximum distance	$d_{\mathrm{MAX}}=882$ km
EIRPD for terminals	EIRPD=−36.82 dBW/Hz
Maximum gain of antenna elements	$G_{R}=5$ dB
Satellite antenna gain-to-noise temperature	G/T=5 dB/K

## Data Availability

Not applicable.

## Appendix A

Remarkably, $R_{ir}$ has embedded the localization information of those signals that have been generated with the repetition pattern $C_{i}$. To prove it, it is useful to realize that if:

(A1) $$E\left\{x_{k}C_{i}C_{j}^{H}x_{k}^{H}\right\}=0,$$

for i≠j, then (20) can be simplified as follows:

(A2) $$R_{ir}=\sum_{k\in N_{i}}E\left\{x_{k}C_{i}C_{i}^{H}x_{k}^{H}\right\}h_{k}h_{k}^{H}.$$

In notation terms, the set $N_{i}$ gathers the index of those users that satisfy $p_{k}=i$. Interestingly, OFDM and SC-FDM waveforms fulfil (A1) when all the carriers are active, as the rows of the inverse discrete Fourier transform (IDFT) matrix are orthogonal. In conclusion, when (A1) holds true, the optimal beamvectors lie within the subspace spanned by $\left\{h_{k}|k\in N_{i}\right\}$. Another interesting result is that $h_{i}$ and $h_{j}$ are asymptotically orthogonal when the number of antennas tends to infinity [28]. Hence, for large scale antenna array systems, any vector that belongs to $\left\{h_{k}|k\in N_{i}\right\}$ solves (21). Closely analyzing (22) we can also resolve that, under the orthogonality conditions, the MV beamformer is proportional to the array vector (8) in the direction of the user of interest. The immediate consequence that results from the orthogonality is that the vector that fulfils (16) is proportional to the MV beamformer.
